# Structural Behavior of Large-Scale Hollow Section RC Beams and Strength Enhancement Using Carbon Fiber Reinforced Polymer (CFRP) Composites

**DOI:** 10.3390/polym14010158

**Published:** 2021-12-31

**Authors:** Athasit Sirisonthi, Phongthorn Julphunthong, Panuwat Joyklad, Suniti Suparp, Nazam Ali, Muhammad Ashraf Javid, Krisada Chaiyasarn, Qudeer Hussain

**Affiliations:** 1 Department of Civil Engineering, Faculty of Engineering, Naresuan University, Phitsanulok 65000, Thailand; s.athasit@gmail.com (A.S.); pop_civil@hotmail.com (P.J.); 2 Sino-Thai Engineering and Construction Public Company Limited (STECON), Bangkok 10110, Thailand; 3 Department of Civil and Environmental Engineering, Faculty of Engineering, Srinakharinwirot University, Nakhonnayok 26120, Thailand; panuwatj@g.swu.ac.th; 4 Department of Civil Engineering, School of Engineering, University of Management and Technology, Lahore 54770, Pakistan; nazam.ali@umt.edu.pk; 5 Department of Civil Engineering, NFC Institute of Engineering and Fertiliser Research, Faisalabad 38090, Pakistan; ma.javid@iefr.edu.pk; 6 Thammasat Research Unit in Infrastructure Inspection and Monitoring, Repair and Strengthening (IIMRS), Thammasat School of Engineering, Faculty of Engineering, Thammasat University Rangsit, Pathumthani 12121, Thailand; ckrisada@engr.tu.ac.th; 7 Center of Excellence in Earthquake Engineering and Vibration, Department of Civil Engineering, Chulalongkorn University, Bangkok 10330, Thailand; ebbadat@hotmail.com

**Keywords:** reinforced concrete beams, hollow section, carbon, fiber reinforced polymers, ultimate deflection, energy dissipation, strain, cost-benefit analysis

## Abstract

An experimental program was conducted to ascertain the efficiency of Carbon Fiber Reinforced Polymer (CFRP) in enhancing the flexural response of hollow section reinforced concrete (RC) beams. Nine beams were tested under four-point bending in three groups. Beams were categorized to reflect the presence or configuration of the CFRP sheet. Each group consisted of three beams: one with a solid section, one with a square 50×50  mm × mm opening and 1 with 100×100  mm × mm opening. Beams in 1st group were tested in as-built conditions. Beams in the 2nd group were strengthened with a single CFRP sheet bonded to their bottom sides. Configuration of CFRP sheet was altered to U-shape applied to the tension side of 3rd group beams. The inclusion of openings, regardless of their size, did not result in degradation of ultimate load and corresponding deflections. However, cracking loads were found to decline as the opening size increased. Regardless of the opening size and CFRP configuration, ultimate loads of beams increased with the application of CFRP. However, this improvement was limited to the debonding and rupture of CFRP in group 2 and 3 beams, respectively. A comparison in the behavior of group 2 and 3 beams revealed that the application of the U-shape CFRP sheet yielded better flexural performance in comparison with the flat-CFRP sheet bonded to the bottom of beams. In the end, In order to further evaluate the economic and performance benefits of these beams, the cost-benefit analysis was also performed. The analysis showed that the feasibility of the hollow section RC beams is more than the solid section RC beams.

## 1. Introduction

Rehabilitation of existing structures or their individual components has been the hub for the past few decades. This can be ascribed to many reasons stretching from environmental influences, change in loading magnitudes, deterioration caused by earthquakes to upgrades in order to meet the modern design provisions. Unsatisfactory performance of any structure can lead to serious concerns from the perspective of public safety. With many older constructions either lacking to meet newly imposed loading requirements or modern code standards, rehabilitation and repair works have become fundamentals to current research activities. Various strengthening techniques have been developed over the years to achieve the aforementioned tasks.

Efficient retrofitting of existing structures has been proven by various methods. The earliest studies focused on the application of conventional concrete jackets around the deficient part of the component or a component as a whole [1,2,3,4,5]. Extensive work has also been done on the use of steel jacketing [6,7,8,9,10]. As much as these conventional methods work effectively for retrofits, some alarming shortcomings associated with them are still inevitable. Concrete or steel jackets are themselves heavy and impart significant weight to the structure thus increasing load demand on underlying foundations. Further, concrete or steel jackets alter the stiffness of the structure with additional concerns related to the corrosion of steel jackets [11,12]. The Rapid evolution of composite materials in the field of structural rehabilitation is appreciable. Their inherited high tensile strengths, corrosion resistance, lightweight, easy to handle, good fatigue, and less labor cost involved make them an excellent alternative to conventional jacketing techniques [13,14,15,16,17].

Openings in RC members are often provided for reasons like reducing self-weights, electrical supply lines, water and sewerage lines, computer networks, etc., [18,19,20,21]. Altun et al. [22] tested nine RC beams with dimensions 200 mm × 200 mm × 3000 mm constructed with classic steel fiber reinforced concrete (CSRC). They examined the effects of reduced dead weights by providing openings, varied wall thickness, and different ratios of the wall thickness to beam height on ultimate load carrying capacity. It was found that a reduction of approximately 44% in deadweight could reduce ultimate load capacity up to 29%. Alnuaimi et al. [18] compared experimental findings of seven hollow beams with those of seven solid beams. Their beams measured 300 × 300 mm x mm in cross-section to 3800 mm in length. By providing an internal hollow core of 200 × 200 mm, a peripheral wall thickness of 50 mm was obtained. They concluded that the hollow core significantly affected hollow beams’ behavior and could not be overlooked under the combined load of bending, shear, and torsion. Gasham [23] tested six moderate deep RC beams embedded with PVC pipes. Their beams measured 150 × 300 mm x mm in cross-section to 1500 mm in length. Four different PVC pipe diameters were utilized to check their effects on beams’ behavior. They concluded that pipe diameter smaller than 33% of beam’s width had a negligible effect on its performance. For larger pipes, the ultimate strength and stiffness of beams reduced as pipe diameter increased. Alshimmeri and Maliki [24] investigated the effects of vertical reinforcement, opening size, and their orientations on the behavior of six beams each measuring 120 × 180 × 1000 mm x mm x mm. It was concluded that the presence of openings reduced ultimate capacity from 37 to 58% and increased corresponding deflections up to 76%. Murugesan and Narayanan [25] investigated the effect of longitudinal circular opening on the flexural strength of simply supported rectangular hollow beams. They varied the diameter of circular holes as 25, 40, and 50 mm as well as their positions from the top. Flexural mode of failure was observed in all hollow beams. Moreover, a theoretical model was proposed to predict flexural strength and load corresponding to the onset of 1st crack.

It can be concluded from previous experimental findings that the presence of openings inside beams can substantially reduce their ultimate strengths with increased corresponding deflections. To preclude this deficiency, different strengthening schemes have been proposed. Dong et al. [26] carried out flexural strengthening of seven RC beams with different cross-sections, longitudinal reinforcement ratio, and concrete cover. CFRP sheets with different layers were externally bonded to the beams to enhance their flexural strengths. A gain of 41–125% in flexural strength was reported in CFRP retrofitted beams. Khuzaie and Atea [27] studied the behavior of hollow RC T-beams constructed with reactive powder concrete (RPC). RPC was made by adding steel fibers and silica fume to concrete in different quantities. They investigated the effect of different volumetric ratios of steel fibers and silica fume. It was found that an addition of steel fibers by 2% to concrete mix could significantly enhance cracking load and ultimate torque of RPC hollow beams. Moreover, cracking, and ultimate torques were increased up to 184 and 66%, respectively. Vijayakumar and Madhavi [28] strengthened hollow RC beams with different fibers. They found that both compressive and flexural strengths of hollow beams were noticeably improved by the addition of micro steel fibers and nylon fibers with volumetric ratios of 0.3 and 0.1%, respectively.

Chen et al. [29] carried out investigations on the influence of CFRP plates in restoring structural properties of rectangular hollow beams. They tested eight beams under 3-point loading out of which three beams were kept as controlled and five beams strengthened with CFRP plates with and without prestressing. They noticed that failure patterns of repaired beams were similar to those of control beams. Besides, prestressing of CFRP plates resulted in further improvement in the behavior of hollow beams.

There are limited studies on RC beams with hollow sections [26,27]. There is an urgent need to investigate the effect of openings on the structural behavior of large-scale hollow section RC beams. Especially openings of larger dimensions which could significantly reduce the weight and cost of the RC beams. A detailed review of existing literature indicates that so far openings of large dimensions have not yet been studied in RC beams. Therefore, the main objective of this stud was to investigate the effect of two different types of openings on the structural behavior of large-scale hollow section RC beams. Given that openings in RC beams reduce their capacities significantly, the need for their strengthening for optimal performance cannot be overlooked. To date, many studies have been published on the use of different types of FRP to alter the strength of the reinforced concrete members, such as beams and columns [30,31,32,33]. Recently, Mohammed et al. 2020 a systematic review of current practices for the repair of structures using prefabricated composite jackets. The authors conclude that the jacketing systems offer superior properties in terms of corrosion resistance, lightweight and durability compared to conventional repair systems [34]. This paper further aims at investigating fiber reinforced polymer (FRP) application on rectangular hollow beams in enhancing their flexural strengths. Carbon FRP sheets are applied to hollow beams in different configurations to allow for the comparison and selection of the best possible configuration that could restore hollow beams’ flexural capacities up to optimum level. To achieve the research objectives, a total number of nine large-scale RC hollow section beams were constructed and tested under a four-point bending scheme. In order to further evaluate the economic and performance benefits of these beams, a cost-benefit analysis was also performed.

## 2. Experimental Program

### 2.1. Test Matrix

**Figure 1:**
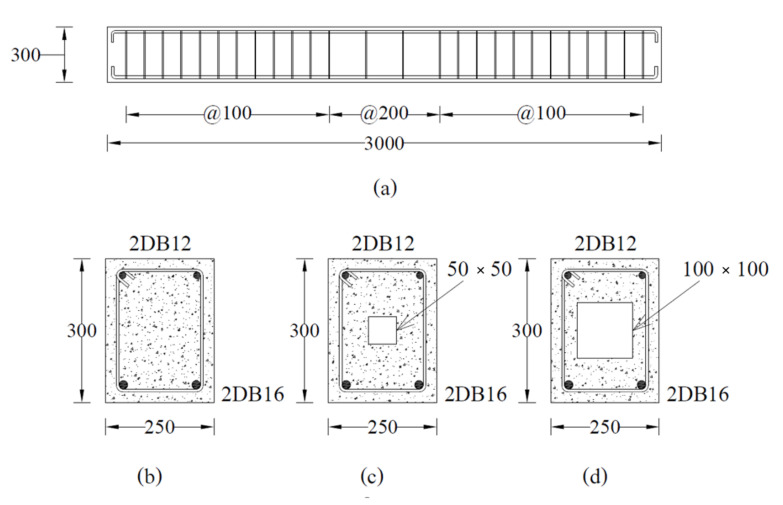
Typical beams in each group (**a**) longitudinal section, (**b**) solid section, (**c**) hollow section HS50, (**d**) hollow section HS100.

**Figure 2:**
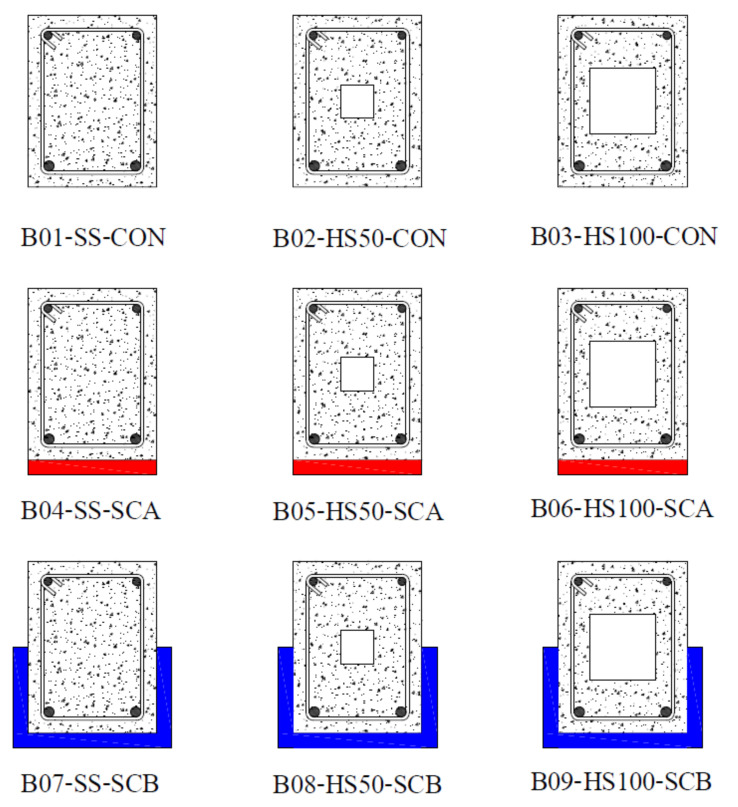
Layout of CFRP sheets.

**Figure 3:**
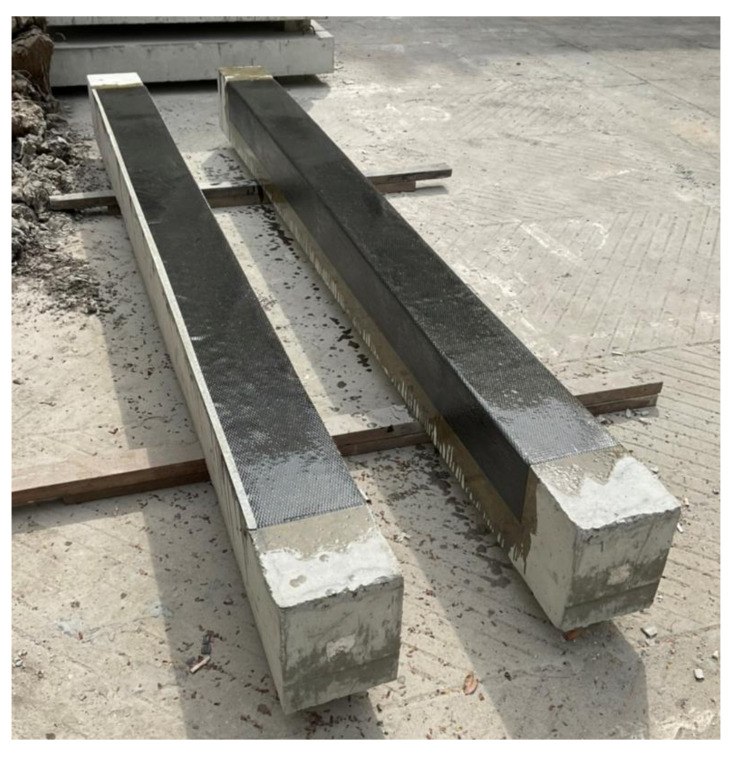
Application of CFRP sheet on bottom of beams (**left**) tension side only (**right**) u-shape sheet.

A total of nine reinforced concrete beams with and without hollow openings were tested in this study. Table 1 presents details of the test matrix adopted. Beams were categorized into three main groups depending upon the presence or configuration of the adopted strengthening scheme. Each group contained three beams: one without any opening, i.e., having a solid cross-section, one with an internal opening of 50×50 mm, and one with an internal opening of 100×100 mm. All openings were made to coincide with the geometric center of beams. Figure 1 presents the typical characteristics of tested beams in each group. To increase the flexural strength of beams with internal openings, group 2 and 3 beams were strengthened with CFRP sheets. Configuration of CFRP sheets was varied in group 2 and 3. Group 2 beams were strengthened with a single CFRP layer applied to their tension sides only thereafter called configuration A (SCA). Beams of group 3 were strengthened with a U-shaped CFRP sheet applied to their tension sides thereafter called configuration B (SCB). The adopted layout of CFRP sheets is shown in Figure 2. Figure 3 presents two beams in inverted position strengthened with CFRP sheet on tension side only (left) and in U-shape (right). Nomenclature of beams was chosen to represent beam’s ID, presence/size of the internal opening, and the configuration of CFRP sheet in chronological order. For instance, B04-SS-SCA represented beam number 4 (B04) with solid section (SS) strengthened with CFRP sheet applied in configuration A.

The reinforced concrete beams were designed following the standard guidelines of ACI standards [35]. However, the hollow sections, i.e., openings were selected in such a way to facilitate the proper concrete cover for steel bars on each face of the beam. Each beam measured 250×300 mm x mm in cross-section to 3000 mm in length. On the tension side, each beam was reinforced with 2 DB-16 (16 mm) steel bars while 2 DB-12 (12 mm) bars were provided in the compression zone. On account of flexural strength as an objective parameter, each beam was provided with sufficient shear reinforcement. Shear reinforcement consisted of round steel bars of 6 mm diameters (RB6) spaced at 100 mm near supports. The spacing of stirrups was increased to 200 mm towards the zone of low shear demand as shown in Figure 1. All beams were incorporated with a concrete cover of 20 mm. To avoid stress concentrations near sharp corners, corners were rounded to a radius of 30 mm to avoid premature rupture of CFRP.

### 2.2. Material Properties

All beams were cast from a single batch of ready-mix concrete. The slump test revealed a value of 120 mm. Standard cylinders were cast as per the recommendations of ASTM C39/C39M-21 [36] and the average 28-days compressive strength was found to be 35 MPa. DB-16 and DB-12 deformed steel bars were used for the bottom and top longitudinal reinforcement, respectively. Vertical reinforcement consisted of round steel bars having 6 mm diameters. Their mechanical properties were found following the protocols of ASTM A615/A615M–20 [37]. Table 2 presents the measured mechanical properties of steel reinforcement. Properties of CFRP sheets were determined as per ASTM D7565/D7565M-10(2017) provisions [38]. The thickness of the CFRP sheet was 1.67 mm with measured ultimate tensile strength and strain as 350 MPa and 1.5%, respectively. The elastic modulus of the CFRP sheet was found to be 250 GPa. Properties of epoxy resin used to bond CFRP sheets with beams were provided by the manufacturer. Provided tensile strength and strain of epoxy resin were 50 MPa and 2.5%, respectively.

### 2.3. Construction and Strengthening

**Figure 4:**
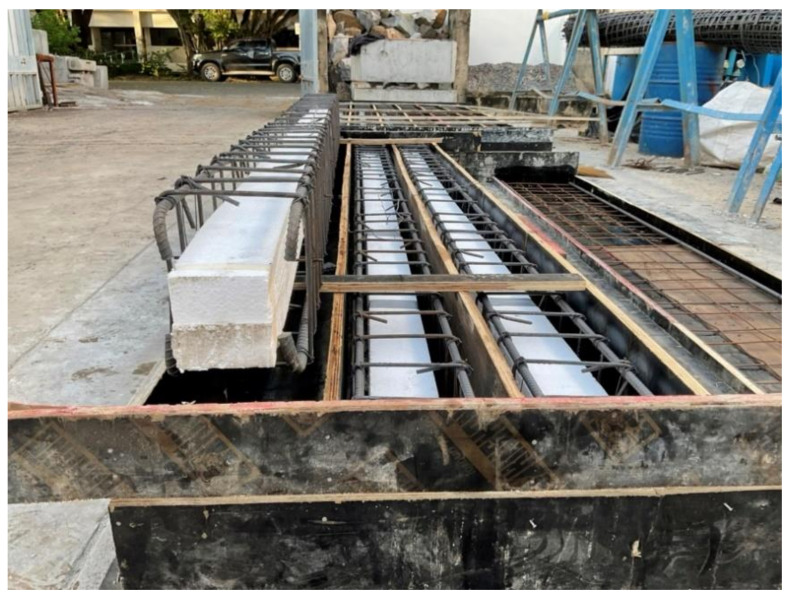
Installation of foam in steel bars.

**Figure 5:**
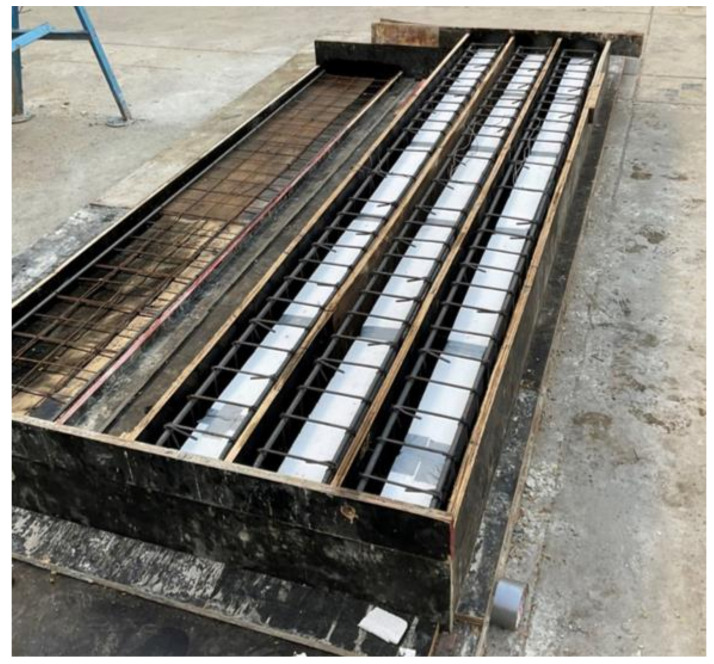
Installation of steel bars in formwork.

The construction of the nine beams was done in the Laboratory. First, the plywood framework was applied for the exact dimensional specifications of the beams. Then, foam of required sizes (50 mm × 50 mm, 100 mm × 100 mm) was cut and placed in the framework (Figure 4 and Figure 5). The wires were used to ensure the stability of the foams so that they should get displaced during the concrete pouring process. The concrete pouring was done using conventional methods of pouring and hand vibrators were used to decrease the vapors and void spaces in the concrete for the sake of compaction. After 24 h the framework was removed and the curing process of the concrete was continued for 7 days. During the curing process, the concrete beams were wrapped with the help of a burlap sack in order to ensure that the proper curing process is done. After the curing process, the sides of the beams were grinned to make them smooth in order to prepare them for the application of the FRP material. The FRP was applied using conventional methods with the help of a hand lap and brush. First, the epoxy resin was applied on the required surface where it was required to apply the FRP, then FRP was applied using a conventional approach (hand lap and brush). After the application of the FRP, again the epoxy resin was applied to make sure that there is an exact bond between the concrete and FRP layers.

### 2.4. Instrumentation & Load Setup

**Figure 6:**
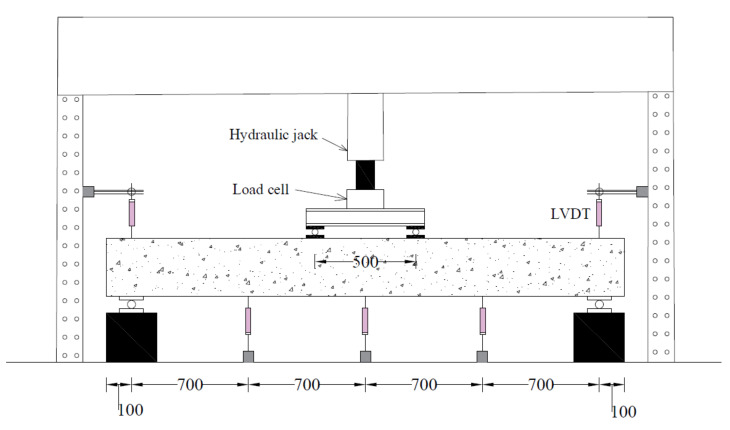
Load setup.

**Figure 7:**
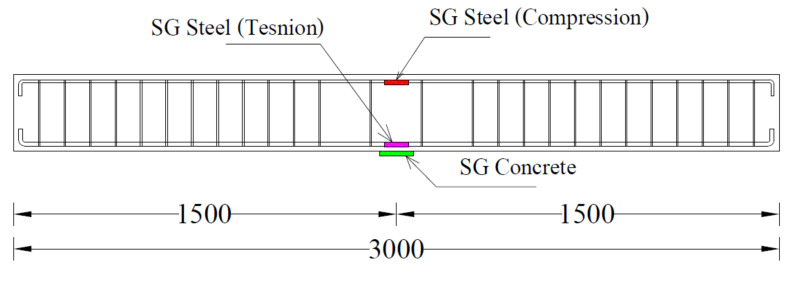
Position of strain gages.

**Figure 8:**
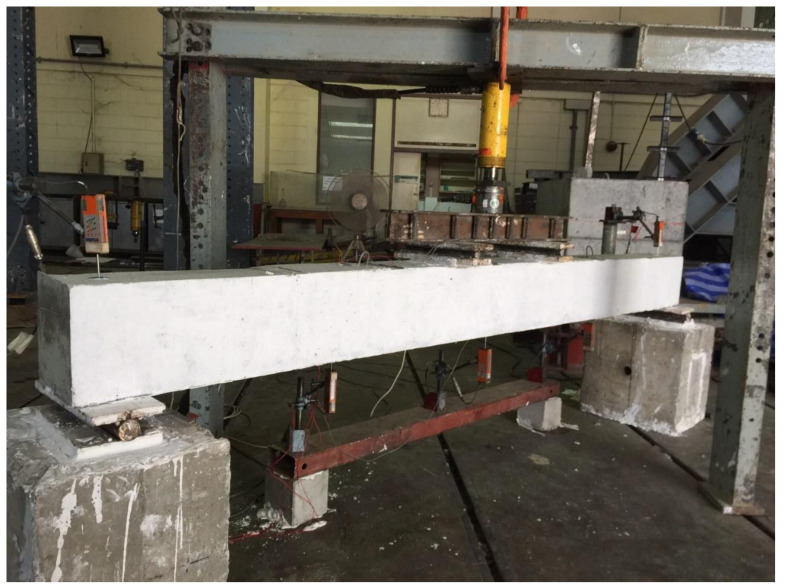
Actual test setup.

A four-point bending loading was applied to each beam as schematically shown in Figure 6. The monotonically increasing load was applied that generated a constant-moment region within the middle 500 mm of beams. Three Linear Variable Differential Transducers (LVDT) were attached at the bottom of each beam to measure their vertical deflections while two LVDTs were attached to each support to measure their uplifts; 5-mm Strain gauges were attached to top and bottom longitudinal reinforcement at midspan as shown in Figure 7. A 60-mm strain gage was also attached to concrete at the bottom side of each beam at its midspan. A load-controlled setup was adopted with load applied using a hydraulic jack whose intensity was measured with a 50 kN load cell placed concentrically underneath it. Figure 8 shows the actual setup during the test.

## 3. Experimental Results

### 3.1. Failure Modes

**Figure 9:**
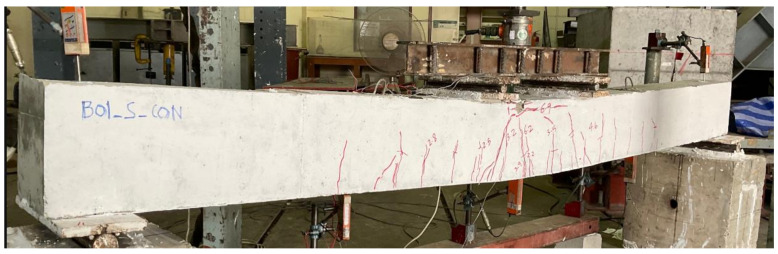
Failure mode of beam B01-SS-CON.

**Figure 10:**
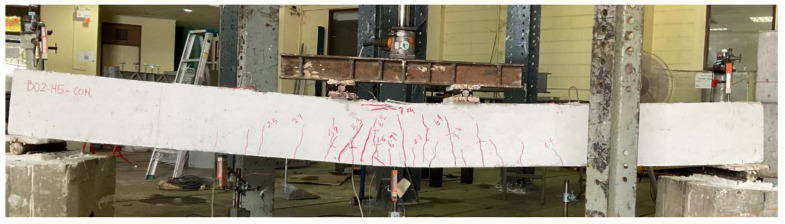
Failure mode of beam B02-HS50-CON.

**Figure 11:**
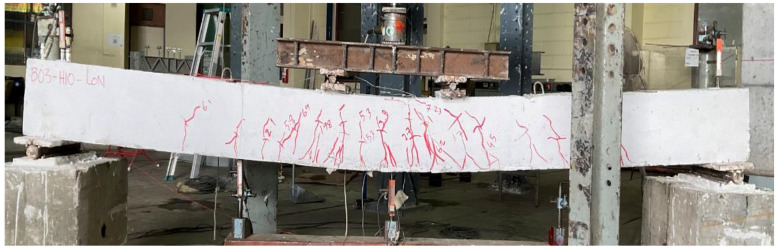
Failure mode of beam B03-HS100-CON.

Beam B01 was a solid section beam without any external CFRP support. This beam behaved in a very ductile way as expected given that it was designed as an under-reinforced section. The cracking load was observed to be 22 kN with the first noticeable flexural crack appearing at its midspan. With the increase in load, further flexural cracks appeared within the constant moment zone as shown in Figure 9. Crushing of concrete at the top of its midspan was observed at a load of 69 kN. The final failure mode was accompanied by the yielding of longitudinal reinforcement and concrete crushing at the top of its midspan. These results are consistent with the previous studies [26,27]. Its failure was a typical representation of ductile failures associated with under-reinforced sections. The second beam in group 1 was furnished with a concentric internal square opening of 50 mm size. This beam behaved in a similar way as the 1st beam B01 (see Figure 10). However, the presence of an internal opening reduced its effective flexural rigidity, thus, enabling an earlier formation of flexural cracks at its midspan. Its cracking load was observed at 16 kN. Concrete crushing was observed at a load of 71 kN that, compared to beam B01, corresponds to a slight increase. Nonetheless, beam B02 experienced a ductile failure analogous to beam B01. An increased opening size in beam B03 did not govern its general behavior as it underwent a ductile failure as well (see Figure 11). However, its cracking load further deteriorated to 15 kN. Ultimate loads of beams B01, B02, and B03 were recorded as 70.81, 72.01, and 74.75 kN, respectively. Another noticeable observation was the occurrence and position of cracks. Beam B03-HS100-CON mobilized more cracks near its supports followed by beam B02 and B01, respectively. In the past, similar cracking patterns have been also reported [18].

**Figure 12:**
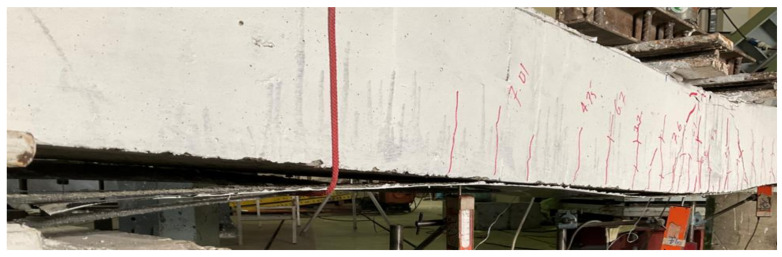
Failure mode of beam B04-SS-SCA.

**Figure 13:**
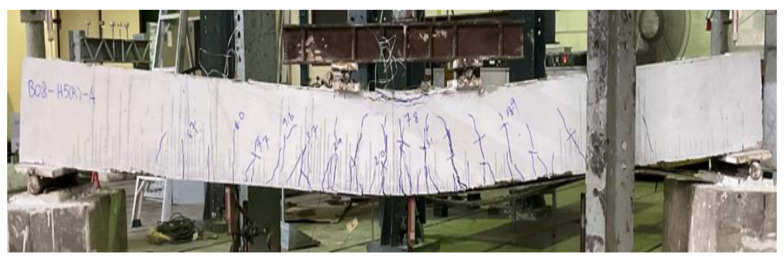
Failure mode of beam B05-HS50-SCA.

**Figure 14:**
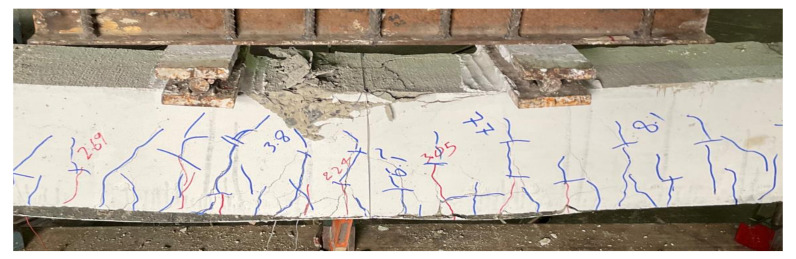
Excessive concrete crushing observed in beam B06-HS100-SCA.

Beams in the 2nd group were strengthened with a single CFRP layer on their bottom sides only. Beam B04, with a solid section, has its final failure shown in Figure 12. With the application of CFRP, the cracking load was slightly increased to 23.1 kN. This beam also performed in a ductile manner. The ultimate failure of this beam is accompanied by sudden de-bonding of the CFRP layer. The onset of debonding initiated at its midspan and proceeded towards supports (see Figure 12). The second beam in this group was B05 that furnished an internal square opening of 50 mm. The failure mode of B05 beam can be seen in Figure 13. Application of CFRP sheet at the bottom side increased its cracking load to 20 kN (i.e., comparing with the cracking load of beam B02). This beam also experienced CFRP debonding at its ultimate failure. Nonetheless, improved ductile behavior was observed as compared to its counterpart control beam in group 1, i.e., B02. Higher concrete crushing was observed at its midspan as compared to that observed in beam B04. Beam B06 exhibited in a similar way to the other two beams in this group. Its failure was ductile limited to CFRP debonding and experienced the highest amount of concrete crushing at its midspan in this group (see Figure 14). In the past, sudden deboning of CFRP from the RS beams has been also reported in many studies [26,29].

**Figure 15:**
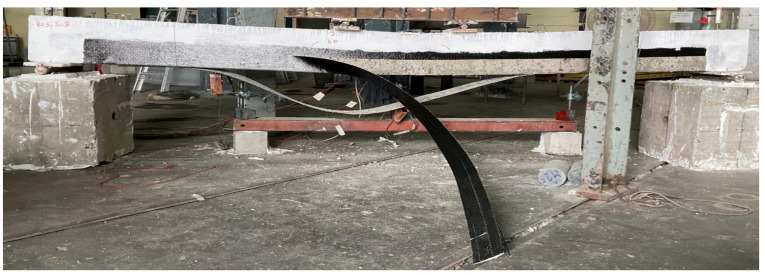
Typical CFRP rupture observed in the 3rd group.

Three beams (i.e., B07, B08, and B09) in the 3rd group were strengthened with a single U-shape sheet (see Figure 2) each. In terms of behavior and failure modes, beams in the 3rd group were almost identical to those in group 2. All beams exhibited a ductile response. However, the CFRP sheet experienced a sudden and explosive rupture near the corners of beams in a transverse direction. It is to be mentioned that CFRP used was unidirectional with its main fibers aligned transverse to the longitudinal axis of beams. Rupture of CFRP fibers was not observed in their longitudinal direction. Though a corner radius of 30 mm was provided along the bottom transverse corners of beams in this group, it could still not prevent stress concentrations resulting in CFRP rupture. A typical failure mode of beams in this group is presented in Figure 15.

### 3.2. Load-Deflection Response

**Figure 16:**
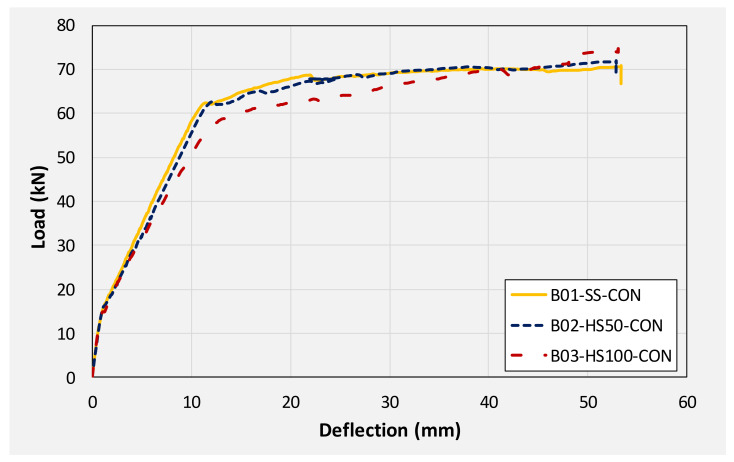
Comparison of load-deflection response of beams in 1st group (no CFRP strengthening).

This section summarizes and compares the experimental load-deflection response of all beams. Firstly, a comparison is made among group 1 beams. Then, subsequent comparisons are made among different beams on the basis of similarities in their geometric configurations. For instance, the load-deflection response of beams B01, B04, and B07 are compared as they share the same section, i.e., without any internal opening. Similarly, the responses of beams B02, B05, and B08 are compared. Finally, the load-deflection response of beams B03, B06, and B09 are compared. Table 3 presents the summary of the key parameters obtained from the load-deflection curve of each beam. Figure 16 presents the load-deflection response of beams in group 1 (i.e., without CFRP strengthening). It can be seen that all three beams exhibited similar initial stiffness up to their cracking loads (though cracking loads slightly varied as mentioned earlier). Up to the yield load, the beam with solid section, i.e., B01 demonstrated the highest stiffness of all followed by beam B02 and B03, respectively. Beam with 100 mm opening experienced the lowest yielding load among its counterpart beams. Nonetheless, the yield deflection of all three beams was comparable.

**Figure 17:**
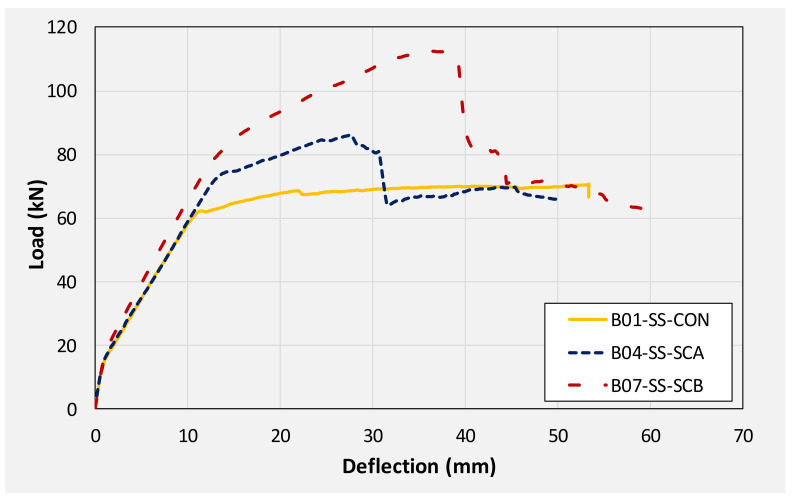
Comparison of load-deflection curves of solid beams.

The application of CFRP, irrespective of its configuration, improved the behavior of beams. Figure 17 compares the load-deflection response of beams B01, B04, and B07. These beams had a solid section with beam B01 acting as control beam while beams B04 and B07 were furnished with CFRP sheets in configuration A and B, respectively. It can be seen that a U-shape CFRP was most effective in terms of improving initial stiffness, load corresponding to yield, and ultimate load. Ultimate loads of beams B01, B04, and B07 were 70.81, 86.22, and 112.81 kN, respectively. This corresponds to an increase of 22 and 59% in ultimate loads sustained by beams B04, and B07, respectively. However, the post-peak behavior of strengthened beams was mainly limited by the performance of CFRP. As shown in Figure 17, sudden drops were observed in load capacities for beams B04 and B07. This is attributed to the sudden debonding and rupture of CFRP in beams B04 and B07, respectively. It is to be mentioned that the rupture of U-shape CFRP was explosive and sudden resulting in larger degradation of load-capacity as compared to beam B04. Another interesting observation was that even after the rupture and debonding of CFRP sheets, load in beams B04 and B07 did not drop below the load carried by the control beam B01. This denoted the importance and effectiveness of CFRP in preventing any underlying damage to beams. Since ultimate loads in CFRP strengthened beams were limited to either CFRP sudden debonding or rupture, corresponding deflections were noticeably lower than that of the controlled beam B01.

**Figure 18:**
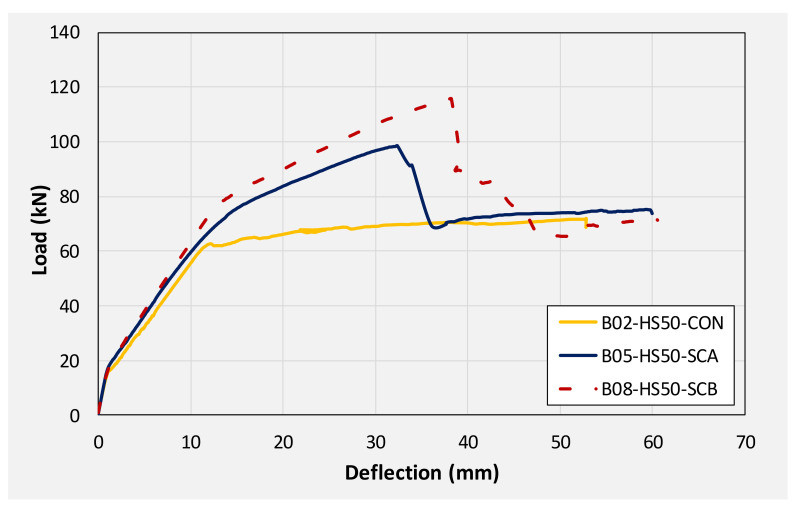
Comparison of load-deflection curves of beams with 50 mm square opening.

Figure 18 compares the load-deflection response of beams with a 50 mm internal square opening. A similar discussion can pertain to beams with 50 mm square openings as it was made for solid section beams earlier. The application of CFRP sheet brought about significant improvements in initial stiffness, yield loads, and ultimate loads. An improvement of approximately 36 and 60% in ultimate loads was observed for beams B05 and B08, respectively. Again, the application of the U-shape CFRP sheet imparted higher improvement to load-capacity as compared to the CFRP sheet on the bottom side only. Analogous to beams in group 1, deflections against ultimate loads were limited ascribing to the sudden CFRP debonding and rupture resulting in an abrupt drop of load to the level of control beam B02.

**Figure 19:**
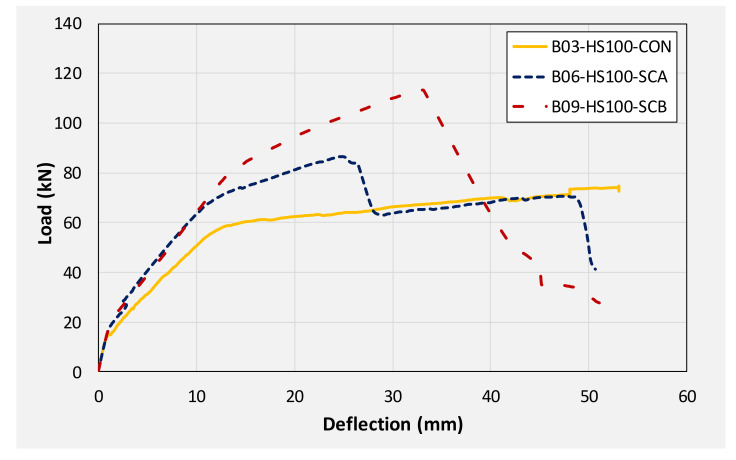
Comparison of load-deflection curves of beams with 100 mm square opening.

**Figure 20:**
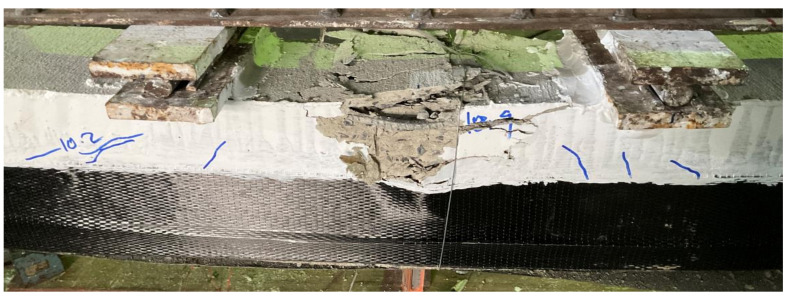
Pronounced concrete crushing observed in beams B06 and B09.

Figure 19 presents a comparison of the load-deflection response of beams with a 100 mm opening. A similar trend as earlier beams was observed in the improvement imparted by CFRP sheets to the load-deflection response. An improvement of 16 and 53% in ultimate loads was observed for beams B05 and B08, respectively. Overall, the U-shape CFRP sheet resulted in more improvement in ultimate loads regardless of the size and presence of the internal opening. Whereas corresponding deflections at ultimate loads were mainly dependent on the behavior of CFRP sheets. Either debonding or rupture of CFRP occurred abruptly causing a sudden drop of load capacities. For solid sections and 50 mm openings, drop in load as a result of either CFRP debonding or rupture was limited and did not fall below the threshold formed by the controlled beam. In the case of beams with 100 mm opening, concrete crushing at midspan was highly pronounced. This caused the load to drop further below the one sustained by controlled beam B03. Typical concrete crushing sustained by 100 mm CFRP strengthened beams is shown in Figure 20.

### 3.3. Steel Strains

**Figure 21:**
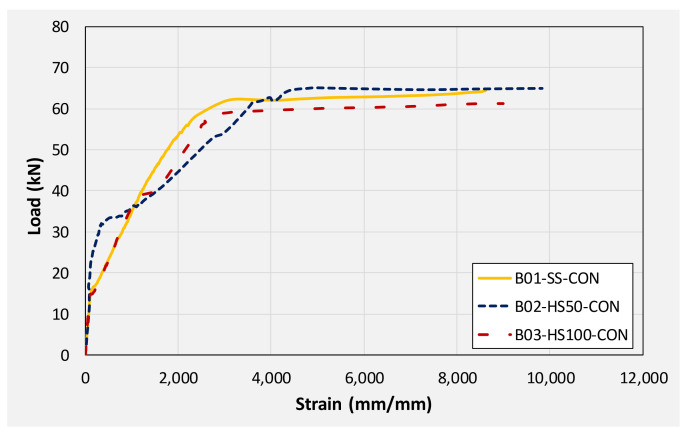
Monitored positive steel strain in beams B01, B02, and B03.

Table 4 presents maximum steel strains monitored using strain gauges attached to the top and longitudinal steel bars. Figure 21 presents strain monitored on bottom longitudinal steel bars at midspan of beams in group 1, i.e., beams B01, B02, and B03. It can be seen that strain gages in all beams reported strains well within their yield plateaus. This was expected in beam B01 having a solid section and designed as under-reinforced. The presence of square openings of size 50 and 100 mm did not transform the ductile response and all the beams were able to develop yielding in longitudinal bars.

**Figure 22:**
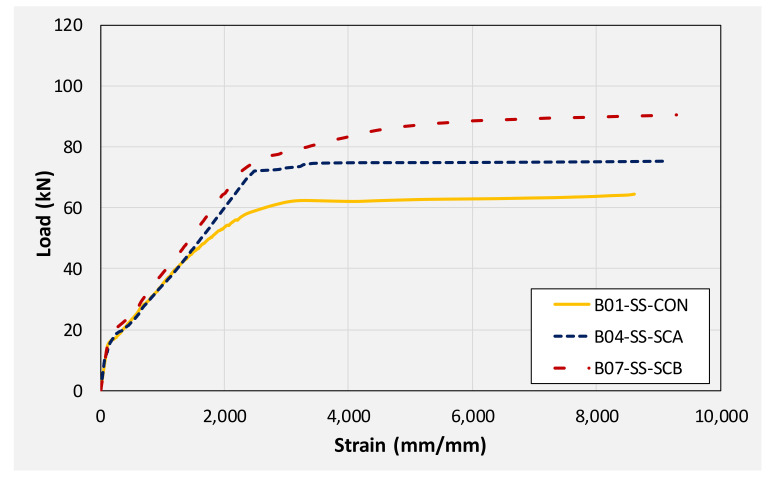
Monitored positive steel strain in solid section beams.

A comparison of positive strains in solid-section beams is presented in Figure 22. It is apparent that the application of CFRP to beams helped mobilize higher strains in bottom steel bars in comparison to the control beam B01. This agrees with load-deflection responses of aforesaid beams. CFRP strengthened beams (B04 and B07) sustained higher loads than their corresponding control beam (B01). Consequently, higher moments were generated within their mid-spans. These higher moments were effectively resisted by bottom longitudinal steel bars which are reflected by their higher measured strains than those of the control beam. Maximum positive strains sustained by tension steel bars of beams B01, B04, and B07 were 8600, 9100, and 9300 μm, respectively.

**Figure 23:**
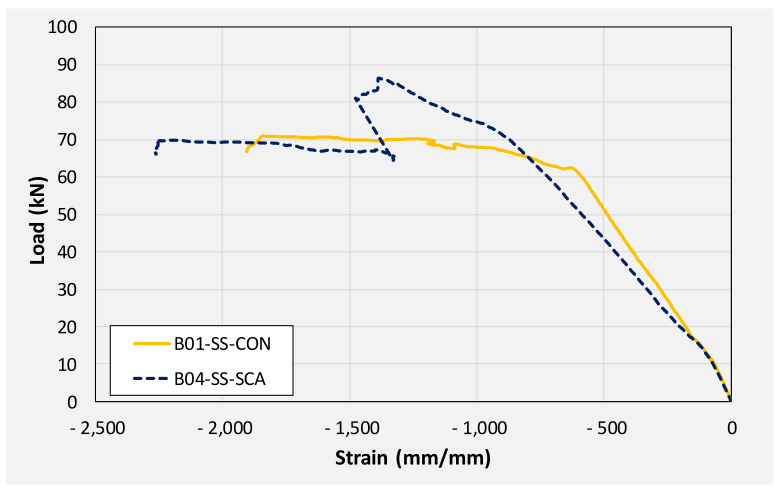
Monitored negative steel strain in solid section beams.

A comparison of compression steel strains monitored on solid section beams is shown in Figure 23. It is to be noted that strain gage mounted on compression steel bar of beam B07 failed prematurely, and reliable strain data could not be extracted. It can be observed that the demand for compression longitudinal steel in CFRP strengthened beam remained similar to that in the control beam. However, compression steel in post-yield regions experienced higher strains as compared to its counterpart steel in the control beam. This can be reflected in that CFRP strengthened beams experienced higher moments in their midspans as compared to the control beams. Consequently, higher compressive stresses were generated in the cross-section above their neutral axes resulting in higher compressive strains. This may be the reason that CFRP strengthened beams sustained larger concrete crushing at their midspans as compared to those experienced by control beams.

**Figure 24:**
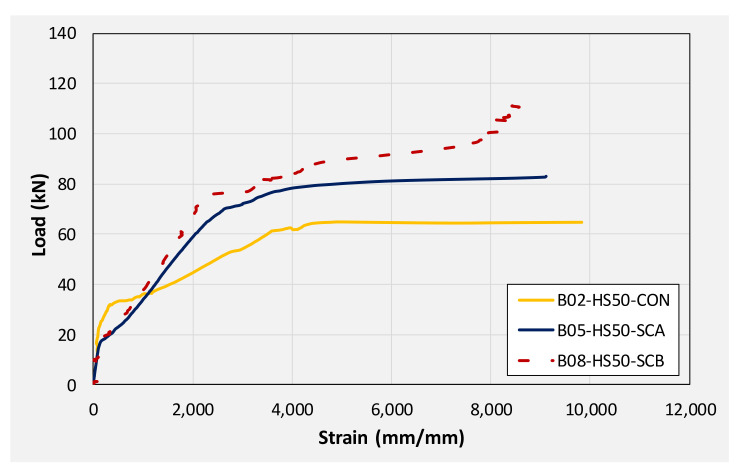
Monitored positive steel strain in beams with 50 mm internal square openings.

**Figure 25:**
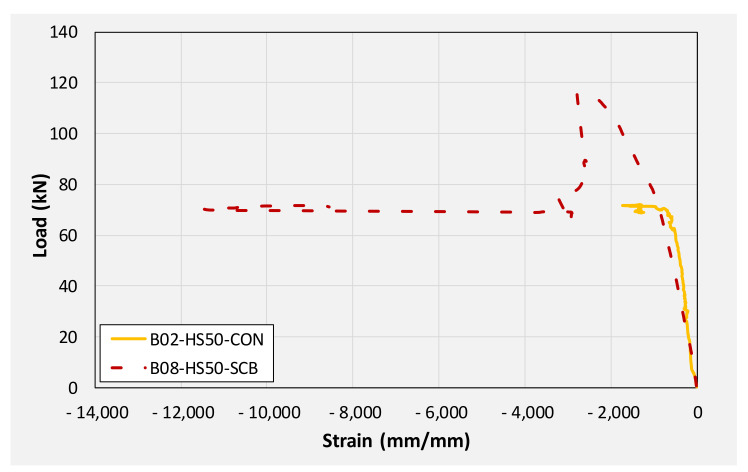
Monitored negative steel strain in beams with 50 mm internal square openings.

Figure 24 and Figure 25 present monitored positive and negative strains, respectively, on longitudinal steel bars in beams with internal 50 mm square opening. Contrary to the maximum strain magnitudes in solid section beams, maximum strains monitored decreased with CFRP strengthening. For instance, beams B02, B05, and B08 recorded 9829, 9115, and 8710 microns, maximum positive steel strains, respectively. This may be ascribed to the efficacy of CFRP in reducing demand on tensile steel slightly. This effect was more pronounced in U-shape CFRP attached to beam B08 as compared to the single CFRP sheet bonded to beam B05’s soffit. The magnitude of negative strains increased as a result of CFRP strengthening due to previously explained reasons. Analogous to solid section beams, a sudden drop in strain-load profiles were seen coincidentally with the sudden drop in peak loads as a result of abrupt debonding of CFRP. Table 4 summarizes peak compressive and tensile longitudinal steel strains in beams with 100 mm internal square openings. It can be seen that the maximum negative strains in beam B03 barely exceeded 1500 microns. However, beams B06 and B09 registered maximum negative steel strains of 20,882 and 10,986 microns, respectively. The magnitude of negative strain recorded in beam B09 was limited to 10,986 microns due to the malfunction of strain gage attributed to large concrete crushing within its vicinity.

### 3.4. Energy Dissipation

The energy absorption capacity of solid and hollow sections RC beams was defined as the area under the curve, i.e., load versus deflection curves (Figure 16, Figure 17 and Figure 18). The energy dissipation for the final step in load versus deflection was ignored as recommended in other studies [39,40]. Table 5 presents the amount of energy dissipated by each beam calculated from the area under their respective load-deflection curves. Comparing energy dissipated by unstrengthened beams, maximum energy was dissipated by the solid section (B01) beam followed by beams with 50 mm and 100 mm openings, respectively. A comparison for dissipated energies by solid section beams revealed that the lowest energy was dissipated by beam B04 (strengthened with CFRP sheet on bottom side only) followed by the unstrengthened beam B01 and beam B07 (strengthened with U-shape CFRP sheet). For beams with 50 mm internal openings, unstrengthened beam B02 dissipated the lowest energy followed by beams B05 (bottom CFRP sheet) and B08 (U-shape CFRP), respectively. A similar trend was also observed for beams with 100 mm internal openings where maximum and minimum dissipated energy bounds were created by beams B09 (U-shape CFRP) and B03 (unstrengthened), respectively.

## 4. Cost-Benefit Analysis

In order to further evaluate the economic and performance benefits of these beams, a cost-benefit analysis is performed. The cost-benefit analysis is also added concerning the feasibility of the design of newly constructed beams. Cost-benefit analysis is important to scale up the design at the industrial level. The results depict some of the interesting findings. This research proposes basically three different benefits over the conventional design of beams; it reduces the weight of the beams, the construction cost is reduced, and the ultimate loading capacity of the beams is improved. As it can be seen from Table 6, the weight of beam B02-HS50-CON and beam B03-HS100-CON is reduced with an approximate amount of 18 Kg (3.33%) and 72 Kg (13.33%), respectively with respect to the control beam (B01). The construction price for these beams is also reported in USD. It can be clearly seen from Table 6, that there is a significant difference of 0.82 USD (3.34%) and 3.27 USD (13.33%) of cost reduction for both beams, B02 and B03, respectively. One of the important findings of this research study, which is very interesting to note that the ultimate load carrying capacity of the proposed modified beams is more than the control beam with an average increase of 1.2 kN (1.66%) and 2.74 kN (5.27%), respectively. Based on these findings, it can be suggested that the feasibility of the newly constructed beams is more as compared with the control beam (B01). Therefore, the newly proposed designs confirm the feasibility of these infrastructural elements (beams) from the sustainability (less concrete), economic (low cost), and performance (load carrying capacity) point of view. In this research study, the use and importance of high-performance beams are evaluated in order to improve the performance mechanism of structures in the face of lower expenses and higher performance as compared with the conventional/control beams. The cost-benefit analysis presented in this research study is imperative and important for the sake of checking the applicability and feasibility of the proposed technique. The proposed mechanism to enhance the performance of the beams would be useless if it is not possible to use this proposed technique on a larger scale in favor of saving the compensating costs associated with production on large scale.

## 5. Conclusions

A total of nine beams were tested in three groups depending upon the presence/configuration of CFRP sheets. Each group was comprised of three beams: one with a solid section, one with an internal square opening of 50×50 mm, and one with an internal opening of 100×100 mm. Group 1 beams were tested in as-built condition. Beams of group 2 were strengthened with a single CFRP sheet bonded their soffits whereas group 3 beams were strengthened with a single U-shape CFRP sheet bonded to their tensile regions. Each beam was tested under four-point bending. Objectives were to study the effect of internal openings on the flexural response of RC beams and the beneficial effects of the CFRP sheet. Further, it was deemed to study the configuration of CFRP sheets yielding optimum results. Based on the results, the following important conclusions can be drawn.

1. Comparing the load-deflection response of unstrengthened beams, cracking load decreased in beam with 50 mm opening compared with that of the solid section beam. It was further reduced in beam with a 100 mm opening. The presence of an opening inside the beam reduces its cracking load which is further reduced as the size of the opening is increased.
2. Unstrengthened beams with 50 (B02) and 100 mm (B03) square openings experienced similar ultimate loads and deflections to those of their counterpart solid section beam (B01).
3. A comparison in ultimate loads sustained by beams with similar openings size or solid section beams revealed that the lowest ultimate loads were recorded for unstrengthened beams. Application of CFRP in both configurations enhanced ultimate loads. However, this improvement was far superior in beams strengthened with U-shape CFRP sheets.
4. Beams strengthened with CFRP sheet bonded to their bottom sides experienced sudden debonding of CFRP at their ultimate loads resulting in an abrupt drop in the load. A similar drop in load was also observed for beams strengthened with U-shape CFRP due to sudden rupture along the beams’ corners. Nonetheless, degradation of peak load, in either case, did not fall below the corresponding load sustained by the control beam. This phenomenon was true for all beams except B09-HS100-SCB which experienced its load degradation lower than that of the control beam. This was attributed to excessive concrete crushing at its midspan resulting in such distinctive behavior.
5. Beam strengthened with CFRP experienced higher compressive longitudinal steel strains. This is an important aspect in the flexural enhancement of beams with internal openings using CFRP. In the absence of inadequate negative longitudinal reinforcement, high compressive stresses above the neutral axis can crush concrete prematurely resulting in brittle failure. A glimpse of such an abrupt load drop was observed in beam B09 that experienced significant concrete crushing.
6. This study has shown that the flexural behavior of the RC solid beams is almost identical to the RC hollow sections beams. In flexural bending, the behavior of RC beams is mainly controlled by longitudinal reinforcement. Further studies are required to clearly understand the presence of the hollow sections on the shear capacity of RC hollow section beams with varying shear span-depth ratios.
7. The cost-benefit analysis showed that the feasibility of the hollow section RC beams is more than the solid section RC beams. Therefore, the out of this study can be further utilized to customize the design of the RC beams and to reduce the cost of the concrete structures.

## Figures and Tables

**Table 1:** Test matrix.

Beam ID	Square Opening Side Dimension (mm)	CFRP Configuration
B01-SS-CON	-	-
B02-HS50-CON	50	-
B03-HS100-CON	100	-
B04-SS-SCA	-	Tension side
B05-HS50-SCA	50	Tension side
B06-HS100-SCA	100	Tension side
B07-SS-SCB	-	U-shape
B08-HS50-SCB	50	U-shape
B09-HS100-SCB	100	U-shape

**Table 2:** Measured mechanical properties of steel reinforcement.

Steel Bar	Yield Strength (MPa)	Ultimate Strength (MPa)
RB-6	250	350
DB-12	400	500
DB-16	420	550

**Table 3:** Key parameters obtained from load-deflection curves.

Beam	Ultimate Load (kN)	Increase in Load (%)	Deflection (mm)
B01-SS-CON	70.81	-	53.41
B04-SS-SCA	86.22	22	27.47
B07-SS-SCB	112.81	59	39.29
B02-HS50-CON	72.01	-	52.84
B05-HS50-SCA	98.52	36	32.35
B08-HS50-SCB	115.47	60	38.26
B03-HS100-CON	74.75	-	53.06
B06-HS100-SCA	86.51	16	25.00
B09-HS100-SCB	113.09	53	32.97

**Table 4:** Summary of maximum strains monitored on longitudinal steel bars.

Beam	Strain (tensile)	Strain (Compression)
B01-SS-CON	8608	1906
B02-HS50-CON	9829	1726
B03-HS100-CON	8991	1501
B04-SS-SCA	9111	2266
B05-HS50-SCA	9115	-
B06-HS100-SCA	7612	20,882
B07-SS-SCB	9299	-
B08-HS50-SCB	8710	11,451
B09-HS100-SCB	8916	10,986

**Table 5:** Energy dissipated by beams.

Beam	Energy Dissipation (kN-mm)
B01-SS-CON	3302
B02-HS50-CON	3240
B03-HS100-CON	2784
B04-SS-SCA	3257
B05-HS50-SCA	4301
B06-HS100-SCA	3293
B07-SS-SCB	4750
B08-HS50-SCB	4301
B09-HS100-SCB	3638

**Table 6:** Feasibility analysis.

Beam	Weight (Kg)	Price (USD)	Ultimate Load (kN)
B01-SS-CON	540	24.53	70.81
B02-HS50-CON	522	23.71	72.01
B03-HS100-CON	468	21.26	74.75

## Data Availability

The data can be availed from the corresponding author upon reasonable request.
